# Profiling the gut resistome to unlock antimicrobial resistance biology and inform clinical risk

**DOI:** 10.1038/s41467-026-75659-5

**Published:** 2026-07-15

**Authors:** Z. Chaudhry, A. Cazares, N. Thomson, RS Heyderman

**Affiliations:** 1 Wellcome Sanger Institute, Cambridge, UK; 2 Institute of Infection, Immunity & Transplantation, University College London, London, UK; 3 The London School of Hygiene and Tropical Medicine, London, UK

**Keywords:** Bacterial genetics, Metagenomics, Metagenomics

## Abstract

Antimicrobial resistance (AMR) is a global crisis, amplified by slow antibiotic development, inadequate surveillance, and limited understanding of resistance gene dissemination. Traditional detection methodologies fail to capture the complexity and interconnectivity of AMR, highlighting the need for novel approaches. Gut resistome profiling using next-generation sequencing enables untargeted detection of AMR genes within microbial communities, revealing their acquisition, transfer and persistence while providing early warning signals for emerging resistance. This review synthesises advances in resistome science, highlights translational opportunities for infection risk prediction and surveillance, and identifies future directions for integrating gut resistome profiling into precision public health and stewardship frameworks.

## Abstract (web summary)

The gut microbiome contains a vast reservoir of antimicrobial resistance genes. In this Review, the authors discuss how gut resistome profiling is advancing understanding of resistance biology while creating new opportunities for surveillance, clinical risk prediction and antimicrobial stewardship.

## Introduction

Since antimicrobial agents became widely available for the treatment of infection in 1945, reports of clinical failure associated with their use have continued to rise; this phenomenon is known as antimicrobial resistance (AMR). A recent global study estimated that 1.27 million deaths were attributable to AMR bacterial pathogens in 2019, which were predominantly driven by six species (*Escherichia coli*, *Staphylococcus aureus, Klebsiella pneumoniae, Streptococcus pneumoniae, Acinetobacter baumannii*, and *Pseudomonas aeruginosa*)^1,2^. If current trends continue, AMR is projected to cause 1.91 million attributable deaths by 2050^3^. These deaths span all age groups and contexts, from infections complicating routine surgery or immunosuppression to increasingly untreatable community-acquired infections.

Historically, AMR understanding has relied on culture-based phenotypic antimicrobial susceptibility testing (AST) of single bacterial isolates, based on consensus cut-offs, to guide clinical decision-making. The development of PCR, Sanger sequencing, and next-generation sequencing (NGS) enabled detection and discovery of genes responsible for AMR (antibiotic resistance genes; ARGs), but until recently these approaches were again largely applied to single cultured isolates with a focus on tracking individual ‘high-risk’ pathogenic species.

However, microbes do not exist in pure culture, but as part of complex, dynamic communities that interact with each other, their host and environment—so called ‘microbiomes’. The human gastrointestinal tract microbiome represents a major ARG reservoir, collectively termed the gut resistome^4^. Notably, ARGs can move independently of bacterial hosts via specialised vectors or mobile genetic elements (MGEs), such as transposons, plasmids and integrons, through horizontal gene transfer (HGT). The collective repertoire of these MGEs within a genome or microbial community is referred to as the mobilome. Metagenomic approaches sequence all genetic material from complex samples, enabling untargeted detection of ARGs and AMR vectors across both pathogens and commensals. Metagenomic approaches using next-generation sequencing (mNGS), through the depth of sequence coverage, provide a more ecologically realistic view of AMR and generate high-resolution data that can be used for early detection and tracking of ARGs and their hosts^5^. Large-scale global mNGS studies have identified over 15,000 non-redundant ARG variants—each defined by less than 99% sequence identity to any other—distributed across human gut metagenomes^5^. This remarkable genetic diversity highlights the vast and underexplored reservoir of resistance potential within the gut, much of which resides in non-pathogenic commensals that are capable of transferring ARGs to clinically relevant species.

**Fig. 1:**
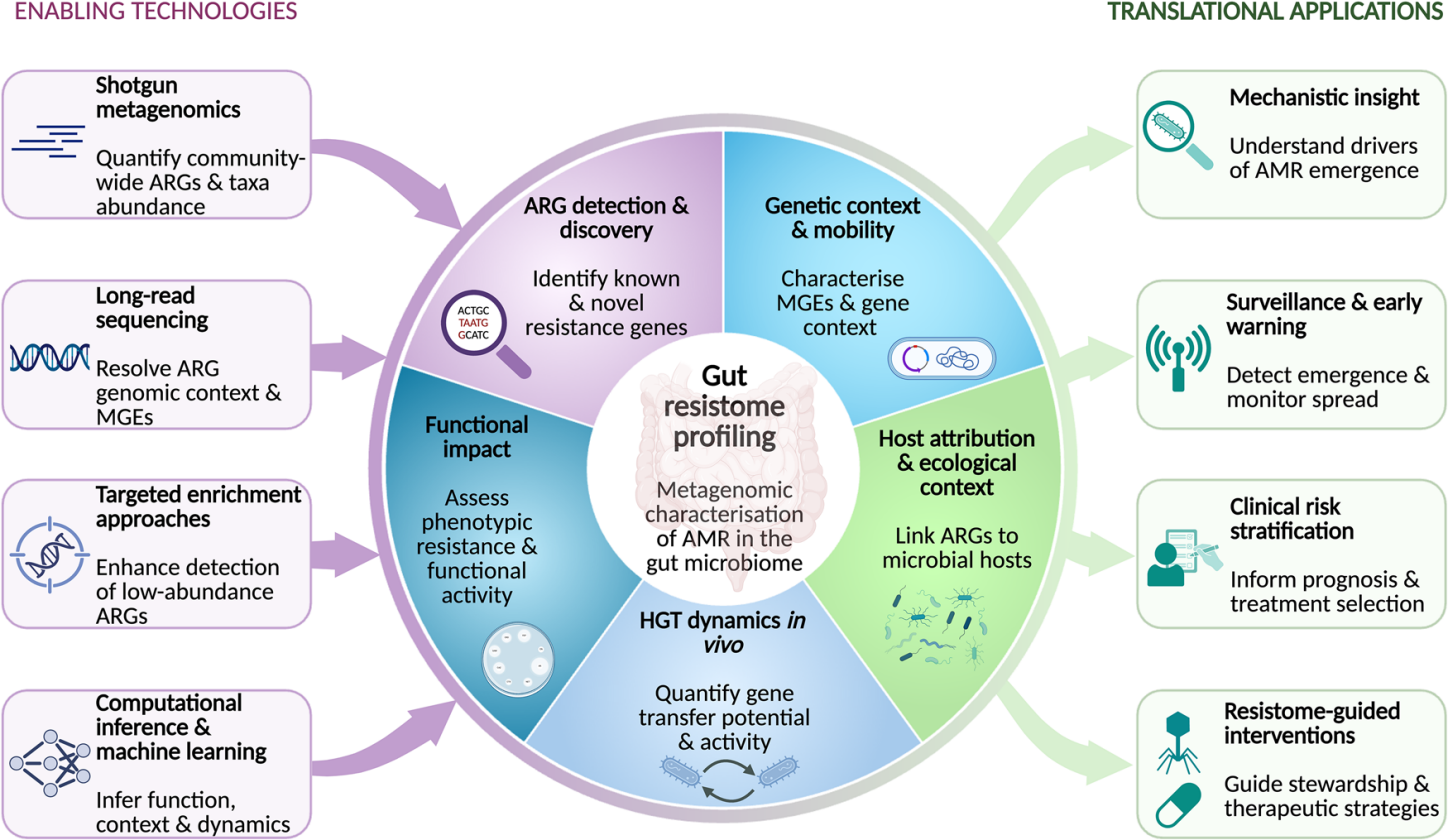
Conceptual framework for gut resistome profiling linking enabling technologies, core analytical domains, and translational applications. Gut resistome profiling combines complementary analytical domains including ARG detection, genetic context & mobility, host attribution, HGT dynamics, and functional impact, to characterise AMR within complex microbial communities. These domains are enabled by distinct methodological approaches, including shotgun and long-read metagenomic sequencing, targeted enrichment, and computational inference, and support downstream applications such as mechanistic insight, surveillance and early warning, clinical risk stratification, and resistome-directed interventions. Created in BioRender. Chaudhry, Z. (2026) https://BioRender.com/9rudhpi.

In this review, we synthesise current advances in gut resistome profiling, outline its contributions to understanding AMR biology, and explore its translational potential in clinical risk prediction and AMR surveillance. We also discuss key methodological challenges and future opportunities for integrating resistome profiling into precision antimicrobial stewardship strategies, highlighting the analytical framework of gut resistome profiling, linking enabling approaches, core analytical domains, and downstream application (Fig. 1).

### Gut resistome profiling to inform fundamental understanding of AMR

The advent of mNGS has transformed resistome profiling, permitting qualitative and quantitative characterisation of all nucleic acids within a sample. Unlike PCR-based approaches, which typically only provide qualitative information, mNGS enables estimation of the relative abundance of specific ARGs within a resistome. In this review, we define relative abundance as gene-length and sequencing-depth normalised measures of ARG signal (e.g. reads per kilobase (kb) per million mapped reads), rather than ratios of ARG reads to total reads, which do not account for differences in gene length.

Such normalisation enables comparison of resistome composition across longitudinal or cross-sectional samples at individual or population level. However, interpretation of relative ARG abundance requires caution, as metagenomic data are inherently compositional. Relative abundance reflects the proportion of ARGs within total sequenced DNA rather than their absolute quantity, and observed changes may arise from shifts in microbial community composition rather than true changes in ARG burden^6^. These considerations are particularly important in longitudinal or interventional studies and highlight the need for complementary approaches, such as quantitative PCR or spike-in standards, to enable absolute quantification^7^.

### High sensitivity of resistome profiling by mNGS

The high sensitivity and untargeted nature of mNGS enables broad detection of ARGs across microbial communities, including those from difficult-to-culture bacterial species. In an early metagenomic study of 162 individuals from three countries, Hu et al.^8^ identified 1093 unique ARGs, illustrating the scale of resistance diversity detectable using mNGS. Most current resistome profiling pipelines rely on alignment to curated reference databases, such as CARD and ResFinder^9,10^, enabling accurate identification of known ARGs. However, this strategy misses novel or highly-divergent ARG variants not represented in reference repositories.

To overcome this limitation, functional metagenomics—which involves cloning random fragments of environmental or microbiome-derived DNA into expression vectors, transforming standardised susceptible bacterial hosts with these constructs and selecting for resistance phenotypes on antibiotic-containing media —can be used to uncover novel ARGs^11^. Using this approach, Sommer et al.^11^ discovered 95 novel ARGs with low sequence similarity to known genes, many associated with MGEs, highlighting the potential for dissemination. More recently, this approach has been extended beyond single-host systems; for example, the DEEPMINE platform uses reprogrammed bacteriophage particles to deliver metagenomic libraries into multiple clinically relevant bacterial species, enabling high-throughput, multi-host discovery of hundreds of previously uncharacterised ARGs, including mobile genes^12^. These findings highlight the value of functional metagenomics for expanding the known resistome while providing insight into the genetic context of ARGs and their potential for dissemination across microbial communities. This contextual information is essential to understanding the mechanisms underlying HGT and identifying ARGs with heightened potential for widespread dissemination across microbial communities, including spread into clinically relevant pathogens and global transmission networks.

### Understanding ARG genetic context

Understanding how selective pressures, such as antibiotic therapy, shape the persistence and spread of ARGs in the gut microbiome remains a major challenge as this depends critically on their genetic context—that is, the physical location of ARGs in a genome or on an MGE and their proximity to genes involved in mobility, replication, or regulation. Short-read NGS approaches are often limited by assembly algorithms that struggle to resolve MGEs, which frequently contain identical, repeated genetic segments that bridge short contiguous segments of DNA (contigs)^13^. ARGs themselves can also form duplicated tandems that are difficult to resolve with short-reads. These limitations reflect broader trade-offs between sequencing technologies in terms of sensitivity, resolution of genomic context, and suitability for different analytical objectives, summarised in Table 1.

**Table 1:** Comparative features and recommended use cases of metagenomic sequencing approaches for ARG profiling

Feature	Short-read	Oxford Nanopore (ONT) long-read	PacBio HiFi long-read	Hybrid approaches (short-read + ONT)
Read length	100–300 base pairs (bp)	Typically 10–100 kb; specialised ultralong protocols can exceed 100 kb	10–25 kb (circular consensus reads)	100 bp – 100 kb (combined short and ONT long reads)
Base accuracy	High	Moderate-high (improving with R10.4 chemistry and newer basecallers)	High (comparable to short reads)	High (short-read accuracy retained; ONT errors corrected by polishing)
ARG detection (known genes)	High sensitivity (read-based alignment)	Moderate (accuracy limitations can affect alignment to reference databases)	High (accuracy supports reliable alignment and variant-level identification)	High
Detection of novel/divergent ARGs	Limited (reference-dependent)	Improved (full gene sequences recoverable; genetic context aids inference)	Improved (high accuracy combined with full gene length enables reliable novel ARG identification)	Improved
ARG genomic context (plasmid/MGE linkage)	Poor (fragmented assemblies)	Strong (spans repeat &, full MGEs)	Strong (spans repeats & full MGEs)	Strong
ARG host attribution	Limited (requires inference)	Improved (long contigs provide linkage signals; native methylation detection with R10.4 chemistry additionally supports epigenetic host linkage)	Improved (long contigs; methylation-based epigenetic signatures additionally enable strain-level host linkage)	Best performance
Assembly quality	Fragmented in complex communities	High contiguity (error rate improving but polishing may be required)	Near-complete to complete circular metagenome-assembled genomes (MAGs) routinely achievable (e.g. hifiasm-meta, metaMDBG)	High contiguity; combines accuracy of short reads with long-range scaffolding
Sensitivity to low-abundance ARGs	High	Lower (coverage-dependent)	Lower (coverage-dependent)	Moderate–high
Bias in complex communities	Lower (read-based methods robust)	Higher (assembly bias toward abundant taxa)	Moderate-high (HiFi-specific assemblers improve performance but coverage depth remains a constraint for rare taxa)	Moderate
Cost & scalability	Low cost per base; highly scalable with established high-throughput platforms	Lower cost per base; multiple flow cell options enable flexible deployment from portable to high-throughput settings	Higher cost per base limits population-scale deployment; best suited to focused high-resolution studies	Higher cost; scalability more limited than short-read alone due to combined data generation and processing requirements; typically reserved for focused research studies
Typical use cases	Large-scale surveillance; ARG abundance profiling; rapid screening	ARG context resolution; plasmid reconstruction; HGT studies; accessible long-read option for clinical and field settings	Near-complete genome recovery; complete plasmid and MGE reconstruction; HGT studies; high-resolution ARG variant profiling	Comprehensive resistome characterisation; high-resolution research studies

*bp* base pairs, *ONT* Oxford Nanopore Technologies, *HiFi* high-fidelity (circular consensus sequencing), *MAG* metagenome-assembled genome.

Long-read sequencing platforms address key limitations of short-read approaches but differ substantially in their characteristics. ONT generates reads typically in the 10–100 kb range, enabling spanning of repetitive regions and full MGE reconstruction, but has historically been limited by reduced base-calling accuracy, with implications for variant-level profiling, though this is improving markedly with advances in flow cell chemistry and base-calling algorithms^14,15^. PacBio HiFi sequencing achieves high accuracy through circular consensus sequencing while maintaining 10–25 kb read lengths, making it well suited to both variant-level ARG identification and recovery of complete circular elements. Single-molecule real-time sequencing has enabled reconstruction of complete extrachromosomal elements from gut metagenomes, including plasmids up to 667 kb, and recovery of previously undescribed MGEs unresolvable by short-read approaches^16^. Purpose-built HiFi assemblers, including hifiasm-meta^17^ and metaMDBG^18^, routinely recover near-complete to complete circular MAGs from complex gut metagenomes, substantially improving plasmid and other MGE recovery.

Hybrid assembly, combining the accuracy and depth of short-read data with ONT long-read scaffolding, offers a practically accessible and well-validated strategy for comprehensive resistome characterisation. In human gut datasets, hybridSPAdes^19^ produced markedly longer and more contiguous assemblies than short-read methods alone, resolving complete plasmid sequences and substantially increasing ARG-encoding contigs and high‑quality MAGs, providing much richer genomic context for ARGs^20^.

Genetic context can also be inferred by analysing genomic regions surrounding ARGs. Wang et al.^21^ analysed multidrug resistance plasmids and showed that ARGs typically occur in dense resistance islands containing multiple ARGs and mobility genes, such as transposases and integron components. Similarly, analysis of sewage resistomes using short-read mNGS compared surrounding DNA regions of different ARGs, providing an estimate of how frequently ARGs had been mobilised between different genetic contexts^22^. This facilitated the identification of highly mobile ARGs, such as *qnrS2*, which confers quinolone resistance, and had transferred across multiple taxa. Applying such approaches to human gut metagenomes could yield important insights into AMR evolution and identify ARGs of public health importance that undergo frequent HGT.

### Ascribing ARGs to their bacterial host

Understanding genetic context reveals how ARGs are arranged within MGEs, but an equally important question is which bacterial hosts carry these genes. This is essential for understanding AMR ecology, HGT, and transmission dynamics. Within the gut, this can help identify commensal and pathogenic species acting as reservoirs of clinically relevant ARGs. However, ARG-host attribution remains a major analytical challenge, particularly with short-read mNGS^23^. Several strategies have been developed to address this, spanning experimental proximity-ligation techniques to computational inference, each with distinct strengths and limitations (Table 2).

**Table 2:** Experimental and computational approaches for assigning ARGs to their microbial hosts in metagenomic datasets

Approach	Primary mechanism for linkage	Best suited for	ARG-host linkage (chromosomal)	ARG-host linkage (MGE)	Strengths	Limitations
Direct host assignment on contigs	Co-localisation of ARG and taxonomic markers on assembled contigs	Well-assembled metagenomes with sufficient contig length	High	Moderate-low	Avoids binning artefacts; faster than full binning workflows; directly captures local genetic context; useful for well-assembled dominant organisms	Limited by assembly fragmentation; mobile ARGs may remain on short or unclassified contigs; plasmids often lack clear taxonomic markers
Read-based assignment	Taxonomic classification of ARG-containing reads using k-mer matching or read-overlapping (e.g. Argo^95^)	Rapid screening of metagenomic datasets; low biomass samples	Moderate	Low	Lowest coverage requirement; no assembly needed; computationally efficient; useful for rapid screening across large datasets	Sensitive to read length, error rate and reference database; short reads may not fully cover ARG, reducing accuracy; poor performance for plasmid-borne ARGs or repeated elements
Binning long reads by methylation motif^23^	Bins long reads by methylation motifs to identify strain-specific signatures	Long-read datasets with sufficient coverage	Moderate-high	Moderate-high	Can enable strain-level resolution; can directly link plasmids to hosts via epigenetic signatures	Requires higher cost long-read sequencing; reduced resolution in highly complex communities as limited by methylation motif uniqueness; less useful for low abundance taxa
Hi-C^96^	Covalently bonds MGEs near to the host genome; sequence of ligation points used for organism ascription	Complex microbial communities; mobile ARGs	Moderate	High	Captures physical DNA proximity in vivo; well-suited for linking plasmids and other MGEs to hosts	Uneven link densities and sequence similarity limits utility for closely related organisms; specialised experimental workflow required
CRISPR-spacer recognition^97^	Evaluates similarity between genomic spacer regions and MGEs	Retrospective host-MGE linkage	Low	Moderate	Highly specific when matches are present; provides historical interaction information; can provide spatiotemporal insights as spacers are acquired chronologically	Limited coverage: cannot capture all ARG-host relationships; not all bacteria have CRISPR systems; requires comprehensive databases for matching spacers
Computational binning^98^	Contigs binned by different features, e.g. sequence similarity or composition features: k-mer frequency; Can be reference dependent or independent	Genome-resolved metagenomics; dominant taxa	High	Low-moderate	Enables organism-level resolution; places ARGs within MAGs, enabling linkage to organism-level gene content, abundance, and ecological dynamics; improved by new deep learning tools (e.g. VAMB^99^, SemiBin2^100^)	Algorithms are computationally intensive (especially if reference independent); biased towards abundant organisms; MGEs often fail to bin correctly; reference-dependent methods limited by database completeness
Single-cell metagenomics^30^	Isolation and sequencing of individual microbial cells	Direct genome reconstruction at single-cell level	High	High	Direct linkage of ARGs and MGEs within the same cell; avoids assembly and binning biases; resolves cell-level genetic context	Technically demanding; amplification bias; currently limited by low throughput and high cost

Recent longitudinal genome-resolved human gut metagenomic studies have shown that antibiotic exposure can enrich highly resistant bacterial populations within specific taxa, particularly Enterobacteriaceae pathobionts, which persist and functionally reshape the gut microbiome following treatment^24^. These findings highlight how defined microbial reservoirs of ARGs may persist beyond antimicrobial exposure, sustaining resistance potential and potentially facilitating onward HGT. Identifying such hosts could inform targeted microbiome-based interventions to reduce ARG persistence or limit opportunities for HGT within the gut.

### Elucidating HGT dynamics

Beyond mapping ARGs and their microbial hosts, resistome profiling can provide insight into the HGT dynamics underpinning AMR evolution within the gut. Long-read sequencing, hybrid assembly, high-resolution binning, and co-barcoding approaches (which link sequences from the same cell via shared molecular barcodes, enabling physical co-localisation of ARGs and MGEs to be inferred) enable reconstruction of HGT events directly from metagenomic data. For example, long-read metagenomic assembly with the myloasm assembler recovered highly contiguous genomes and resolved nearly identical *ermF* ARGs carried on distinct strain‑specific MGEs in the human gut, illustrating how long reads can disentangle ARG movement between closely related backgrounds^25^. Similarly, co‑barcoding-based, genome‑resolved metagenomics can map ARG transfer between distantly related taxa in situ, quantifying thousands of HGT blocks and revealing enrichment of specific resistance functions within host‑associated microbiomes^26^. These approaches enable HGT dynamics to be studied in vivo within complex microbiomes, moving beyond in vitro single-isolate studies that lack ecological context.

Through mNGS and profiling viral DNA/RNA, there is increasing recognition that bacteriophages (phages; viruses that target bacteria) contribute substantially to ARG dissemination through phage-mediated transduction. Network-based analyses of metagenomic datasets consistently identify tailed double-stranded DNA phages, particularly Siphoviruses and Myoviruses, as vectors carrying ARGs conferring resistance to multiple antibiotic classes across bacterial hosts^27^. Comparative analyses across faecal and environmental microbiomes further demonstrate conserved ARG-associated viromes despite substantial differences in bacterial composition, suggesting that phages act as mobile conduits for resistance dissemination across ecosystems^27^. These findings challenge the traditional emphasis on plasmid-mediated ARG transfer and highlight the importance of viral-mediated processes in shaping the gut resistome.

Complementing these approaches, Peng et al.^28^ developed HDMI, a workflow that detects recent HGT events by identifying highly similar sequence regions shared between distinct MAGs. Applied to longitudinal shotgun metagenomes from 338 individuals sampled four years apart, this identified 5644 high-confidence HGT events across 116 gut bacterial species and demonstrated that individual mobile gene pools are highly personalised and shaped by host lifestyle factors including medication use. These advances collectively establish metagenomic resistome profiling as a key tool for moving from descriptive surveillance toward a mechanistic understanding of AMR evolution, defining how, when, and between whom ARGs are mobilised within the gut ecosystem^29^.

### Emerging single-cell and spatial approaches

Bulk mNGS provides a powerful overview of resistome composition but relies on DNA extracted from homogenised microbial communities, obscuring cellular and spatial relationships between ARGs and their hosts. This limits resolution of fragmented assemblies and confident assignment of MGEs to specific bacterial hosts. Emerging single-cell and spatially resolved approaches offer complementary strategies to overcome these limitations by preserving this contextual information, enabling more direct interrogation of ARG-host associations and HGT within complex gut microbiomes.

Single-cell metagenomics isolates individual microbial cells prior to genome amplification and sequencing, allowing reconstruction of genomes without reliance on assembly-based binning^30^. Recent advances have enabled high-throughput single-cell genome recovery from the human gut microbiome. For example, Microbe-seq generated thousands of single-cell genomes from human gut samples, enabling strain-level reconstruction across diverse taxa and identification of MGEs shared across microbial lineages^30^. Such approaches provide a direct route to linking ARGs to host genomes and may enable reconstruction of HGT networks across microbial communities.

Beyond improving host attribution, single-cell metagenomics can address biological questions that bulk approaches cannot. Direct sequencing of individual cells provides empirical evidence that an ARG and a given host taxon physically co-occur, rather than inferring host assignment from compositional similarity in mixed assemblies, where an ARG-bearing contig may be associated with a taxon that does not in fact carry the gene. This approach also enables detection of heteroresistance, the co-existence of resistant and susceptible cells within a clonal population, which is invisible to bulk sequencing and has been shown to drive treatment failure in clinical cohorts^31^. Although whole-genome amplification bias and throughput constraints currently limit application to small community surveys^32^, single-cell methods applied longitudinally could ultimately resolve the cellular dynamics of HGT, including the bacterial subpopulations within which mobile ARGs first emerge.

Spatial metagenomic methods aim to preserve the physical organisation of microbial communities within samples, which is lost during standard extraction workflows. Richardson et al.^33^ described Split-And-pool Metagenomic Plot-sampling sequencing (SAMPL-seq), a high-throughput spatial metagenomic method that captures micron-scale gut subcommunities through split-and-pool barcoding. Applied to the healthy human gut microbiome, SAMPL-seq identified reproducible co-localised taxa and spatial hubs dominated by Bacteroidaceae, Ruminococcaceae and Lachnospiraceae, and showed that these local structures can rearrange in response to dietary perturbation. Although shallow per-compartment sequencing depth currently limits detection of low-abundance community members^33^, including ARGs, such approaches could help define the micron-scale ecological niches in which ARG exchange is most likely to occur. They could also test whether spatial co-localisation in biofilm-like microenvironments, rather than overall taxonomic abundance, is the dominant determinant of HGT frequency in vivo. Combined with single-cell sequencing, spatial methods could ultimately link ARG transfer events to specific physical interfaces between donor and recipient cells.

### Enrichment techniques for improved ARG detection

Resistome profiling is limited by the low abundance of ARGs in shotgun metagenomic data, typically comprising <0.5% of reads in the human gut microbiome^8^. This complicates interpretation in longitudinal studies, where distinguishing de novo acquisition from expansion of low-abundance ARGs under antimicrobial selection is challenging.

Targeted enrichment approaches, including amplicon-based and hybrid capture methods, have been developed to improve sensitivity. Amplicon-based approaches use targeted PCR amplification of predefined ARG sequences and can increase on-target reads by up to 9.2 × 10^4^-fold compared to shotgun mNGS^34^. These methods are highly sensitive, particularly in low-biomass samples, but are limited to known ARGs and constrained by primer design, potentially missing novel or divergent variants.

Hybrid capture methods use libraries of biotinylated probes, often derived from curated databases such as CARD, enabling broader ARG detection. Probe sets targeting tens of thousands of sequences across thousands of ARG families have improved detection breadth, with recent designs encompassing over 34,000 sequences targeting more than 4600 ARGs^35^. Hybrid capture can achieve >600-fold enrichment while also capturing flanking genomic regions, providing insight into MGEs and HGT. This is particularly valuable in complex environments such as the gut microbiome, where probe sets can be iteratively updated as new ARGs are characterised, though probe synthesis and hybridisation add cost and workflow complexity relative to shotgun approaches.

Target-enriched long read sequencing (TelSEQ) extends this approach by combining probe-based enrichment with long-read sequencing^36^. Applied to diverse metagenome types including human stool, TelSEQ achieved >1000-fold ARG recovery gains over untargeted short-read sequencing, with identification of flanking MGEs and cargo genes indicating HGT potential for low-abundance ARGs of public health importance^36^.

### The challenges of ARG reference databases

Resistome profiling results vary depending on sequencing data, analytical approach, and the reference databases used for ARG and MGE identification. A key challenge lies in how ARGs are defined. Clinical frameworks typically restrict ARGs to genes associated with resistance in human pathogens, whereas metagenomic and environmental studies often adopt broader definitions that include genes with the potential to confer resistance when expressed. This distinction has been the subject of recent discussion^37^ and is reflected in studies that apply different inclusion criteria when defining ARGs^38^, for example excluding gene classes such as efflux pumps where links to clinically-relevant resistance may be indirect. These differences influence database composition and contribute to variability in resistome profiling.

Benchmarking studies have highlighted trade-offs between assembly- and alignment-based approaches^39,40^. Assembly-based methods generally provide higher precision in gene reconstruction and enable contextual analysis but may reduce sensitivity for low-abundance ARGs due to minimum coverage requirements. In addition, assemblies are particularly prone to fragmentation around ARGs, because ARGs often reside within repetitive MGEs and occur in multiple genomic contexts across diverse species, which leads to contigs of uncertain origin that complicate downstream annotation and risk assessment^39^. Alignment-based approaches offer greater sensitivity for detecting known ARGs but are dependent on user-defined identity thresholds; stringent thresholds improve specificity but reduce sensitivity, whereas relaxed thresholds increase detection at the expense of false positives. These methods may also struggle to distinguish closely related ARG variants, such as β-lactamase families, where small sequence differences alter gene classification, leading to inconsistent annotation rather than absence of detection. Furthermore, alignment-based approaches generally lack the ability to resolve genomic context, limiting inference of ARG localisation on MGEs or within specific host taxa. Assembly-based workflows can mitigate some of these issues by resolving full-length sequences and their surrounding genetic environments, while alignment-based methods remain well suited to sensitive, rapid detection of known ARGs across complex metagenomic samples.

Inconsistencies across reference databases, including differences in curation standards, nomenclature and scope, further complicate interpretation. Importantly, ARG reference databases differ in scope and intended use. For example, ResFinder and AMRFinderPlus focus on acquired resistance determinants and clinically relevant mutations, whereas CARD adopts a broader ontology-driven framework that includes intrinsic resistance and homologous gene families. Specialised resources such as ARKbase prioritise WHO priority pathogens. These differences can lead to variation in ARG detection and interpretation across studies (Table 3).

**Table 3:** Commonly used ARG reference databases and their characteristics

Database	Scope	Key Features	Strengths	Limitations	Typical use case
CARD^101^	Broad (intrinsic + acquired ARGs)	Ontology-driven, curated, includes resistance mechanisms	High curation quality; mechanistic annotation	Broader inclusion of homologous sequences may reduce specificity in some applications	Comprehensive resistome profiling; mechanistic studies
ResFinder^10^	Primarily acquired ARGs + some mutations	Acquired ARGs + chromosomal point mutations via integrated PointFinder	High specificity for acquired resistance	Limited coverage of intrinsic genes	Clinical surveillance; pathogen genomics
NCBI AMRFinderPlus^102^	Acquired genes + resistance mutations	Integrated with RefSeq; includes point mutations	Standardised, widely used	Overlap with CARD; less flexible ontology	Clinical and public health pipelines
MEGARes^103^	Hierarchical classification of ARGs	Structured ontology for abundance profiling	Good for metagenomics quantification	Less emphasis on allele-level resolution	Resistome abundance profiling
ARKbase^104^	WHO priority pathogens	Integrated AMR knowledgebase covering ARGs, virulence factors, drug targets, AST profiles, protein interactions and ML models for WHO priority pathogens	Comprehensive pathogen-focused AMR annotation; integrates genomic, structural and clinical data; covers 2024 WHO priority pathogen list	Not designed for metagenomic ARG profiling or resistome abundance analysis; narrower pathogen scope limits use in broad environmental or gut resistome studies	Translational AMR research; drug target identification; pathogen-focused clinical and public health studies
PLM-ARG^44^	Protein language model-based ML approach	Trained on >28,000 ARGs across 29 resistance categories; enables detection of divergent ARGs with low sequence similarity to known references	Improved detection of novel and divergent ARGs; less constrained by reference database completeness	Dependent on training data quality; limited interpretability; lacks the regular versioned updates of curated sequence databases	ML-based ARG prediction; detection of divergent and novel ARGs

Variation in ARG definitions also affects the inclusion of resistance-associated mutations and regulatory elements. Some databases incorporate point mutations (e.g. *gyrA*, *rpoB*) and regulators (e.g. *marR*, *ramR*) that modulate resistance phenotypes. While this can provide a more comprehensive representation of resistance potential, it complicates interpretation as these elements are often context-dependent and may not confer resistance in isolation^5^.

Database quality is another key limitation. Many resources contain entries supported by variable levels of experimental validation and may include misannotated or incompletely characterised ARGs^41^. These discrepancies can affect both assembly- and alignment-based workflows, leading to divergent gene calls and antibiotic class assignments and reducing reproducibility. Furthermore, genotype-to-phenotype concordance remains incomplete even in single-pathogen sequencing: in one recent analysis, database-based resistance predictions failed regulatory accuracy thresholds for all seven antibiotics tested, with substantial phenotypic resistance unexplained by detected ARGs^42^—a gap that is likely wider in complex metagenomic settings. Transparent reporting of database choice and parameter settings, alongside sensitivity analyses using multiple approaches, can help mitigate these issues.

These challenges have driven the development of ML-based approaches for ARG detection. Unlike rule-based methods, ML models can capture complex sequence patterns and identify ARGs with low similarity to known references. Tools such as DeepARG^43^ and more recent approaches, including protein language model-based methods (e.g. PLM-ARG^44^), use ML to distinguish ARGs from non-ARG sequences based on sequence composition and functional features^45^. These approaches improve sensitivity for detecting remote homologues and novel variants and are less constrained by database fragmentation. However, they require careful validation, depend on training data quality, and may lack interpretability, limiting clinical applicability. As such, ML-based methods are best used alongside traditional pipelines and expert curation.

### Using resistome profiling to characterise intrinsic resistance

Given their potential to disseminate across microbiota, resistome studies often prioritise mobile ARGs. However, characterising non-mobile chromosomal ARGs that confer clonally-inherited intrinsic resistance can reveal fundamental insights into AMR evolution. These genes typically encode core physiological features, including outer membrane permeability barriers in Gram-negative bacteria and multidrug efflux systems, which collectively limit intracellular antibiotic accumulation^46^. ‘Knocking-out’ these genes can render their bacterial hosts highly susceptible to multiple classes of antibiotic, underscoring their role in baseline resistance phenotypes^47^.

Beyond their mechanistic importance, intrinsic ARGs also inform the evolutionary origins of AMR. Phylogenetic and metagenomic analyses demonstrate that many clinically relevant ARGs predate the antibiotic era and are widespread in environmental reservoirs. For example, vancomycin resistance elements have been identified in environmental microbes, and substantial sequence homology exists between ARGs in soil bacteria and those detected in the human gut microbiome^48,49^. These observations support the view that AMR is an ancient and ecologically embedded trait shaped by long-term selective pressures.

Incorporating intrinsic ARGs into resistome profiling frameworks therefore extends analysis beyond transmissible resistance to include the underlying structural determinants of antimicrobial susceptibility. This broader perspective may reveal conserved vulnerabilities that are overlooked when focusing solely on mobile elements, with potential implications for antimicrobial development. Accordingly, both intrinsic and mobile ARGs should be considered in comprehensive resistome studies and surveillance strategies.

### Resistome profiling to study ARG fitness cost and persistence

Resistome analysis provides a valuable framework to study the fitness costs associated with AMR and the strategies bacterial hosts use to mitigate these costs. Acquisition of resistance elements, particularly multi-resistance plasmids, often imposes a fitness burden by reducing growth or competitive ability in the absence of antibiotic selection^50^. However, bacteria can develop compensatory mutations, often in chromosomal housekeeping genes, that restore fitness while maintaining AMR^51^. Additionally, plasmids carrying both ARGs and virulence determinants may persist through co-selection, as virulence factors may confer selective advantages that promote maintenance of ARGs even in the absence of antibiotic pressure^52^.

Resistome profiling provides a powerful means to observe these compensatory evolutionary dynamics in situ. This has important implications for antimicrobial stewardship, which often assumes that removal of selective pressure will lead to a decline in resistance. While in vitro studies, typically involving single host-plasmid pairs, have demonstrated compensatory adaptation^53,54^, they do not capture the ecological complexity of the gut microbiome, where diverse bacterial populations and MGEs co-evolve.

Recent longitudinal metagenomic studies provide empirical evidence that challenges the assumption that resistance will decline on removal of antibiotic pressure. Yaffe et al.^55^ tracked the gut resistome of 60 healthy individuals following a five-day course of ciprofloxacin and identified convergent selective sweeps of *gyrA* across multiple taxa. Notably, these resistance-associated variants persisted for over 10 weeks without detectable fitness cost. This study also uncovered ‘soft sweeps,’ where multiple resistant *gyrA* variants rose in frequency simultaneously, highlighting the diversity of adaptive trajectories under shared selective pressure. These findings demonstrate that resistance can be stably maintained even in the absence of ongoing selection, complicating predictions of resistance decay. Importantly, they illustrate how longitudinal resistome profiling can resolve the dynamics of AMR evolution and persistence in vivo.

These observations also bear on a longstanding question of whether resistance represents a transient stress response that decays under permissive conditions, or a stably integrated component of microbial genomes. The persistence data from longitudinal human studies increasingly support the latter. If compensatory adaptation routinely offsets the fitness cost of resistance within weeks of acquisition, the assumption that resistance frequencies will decline once antibiotic pressure is removed is difficult to support at the level of the gut microbiome. Resistome profiling, by tracking ARG frequencies, MGE architectures, and host genomic backgrounds simultaneously, provides the empirical resolution needed to test how often compensation occurs across the gut microbiome.

Quantitative, longitudinal data on allele dynamics, strain turnover, and MGE persistence can also inform evolutionary and epidemiological models of AMR. Recent work tracking the gut plasmidome during antibiotic exposure showed transient depletion and subsequent recovery of ESBL-producing *E. coli* and associated ARGs^56^, illustrating the kinds of resilience patterns that need to be captured if stewardship strategies are to predict resistance persistence accurately.

### Gut resistome profiling for public health surveillance

AMR surveillance has traditionally relied on culture-based AST, which fails to capture the majority of difficult-to-culture gut bacteria, particularly obligate anaerobes, that may act as reservoirs of resistance for pathogens such as Enterobacterales^57^. Consequently, emerging resistance may remain undetected until clinically manifest. mNGS enables culture-independent profiling of the gut resistome, detecting ARGs across both pathogenic and commensal taxa, and supporting earlier identification of emerging threats. Importantly, mNGS can also resolve the genetic context of ARGs, including their association with MGEs, allowing surveillance to move beyond tracking individual genes or strains towards tracking the vectors that mediate rapid dissemination of resistance. This shift towards monitoring high-risk MGEs represents a key advantage of resistome-based approaches and may enable more proactive, transmission-focused public health interventions. In principle, integrating resistome composition with genomic context and longitudinal sampling may also enable prediction of which resistance elements are likely to persist or disseminate.

Population-level resistome surveillance has already been demonstrated at scale. In a landmark wastewater-based study spanning 101 countries, Munk et al.^22^ used mNGS to characterise the global distribution of ARGs, identifying distinct regional patterns and hotspots. These findings highlight the feasibility of large-scale resistome surveillance across diverse settings, and its potential to generate actionable insights into global ARG dissemination.

At the individual level, longitudinal metagenomic studies have been used to track ARG acquisition and transmission. Worby et al.^58^ analysed stool samples from travellers before and after international travel, demonstrating substantial increases in ARG burden, particularly following travel to Southeast Asia. These findings provide evidence of real-world resistome perturbation and transmission, and illustrate how targeted resistome surveillance could be used to identify high-risk exposures and populations.

Together, these studies demonstrate the potential of gut resistome profiling to transform AMR surveillance. By capturing ARGs across the full spectrum of microbial diversity, including difficult-to-culture taxa and MGEs, mNGS supports a shift from reactive detection to proactive surveillance of resistance emergence and spread. Although challenges remain, including cost, standardisation, and interpretation, ongoing advances in sequencing and analytics are likely to facilitate integration into surveillance frameworks. Integration with existing genomic surveillance platforms, such as pathogen whole-genome sequencing and wastewater monitoring, may further enhance early warning systems for AMR emergence. Targeted applications, such as travel-associated screening, high-risk patient monitoring, and One Health surveillance, represent promising entry points for implementation, and may help intercept resistance threats before they spread more widely.

### Gut resistome profiling for patient management

#### Clinical risk prediction

Gut resistome profiling offers a promising route to identify patients at risk of adverse clinical outcomes, particularly infections with multidrug-resistant organisms (MDROs). While numerous studies have linked gut microbial dysbiosis to disease states^59,60^, fewer have examined the resistome as an independent marker of clinical risk.

Resistome features have been associated with chronic disease risk in observational cohort studies of asthma and type 2 diabetes^61,62^, though in both cases the resistome signal appeared to track underlying taxonomic structure, particularly *E. coli* dominance and gut microbiome immaturity^61^, rather than provide independent predictive value. These findings illustrate the central importance of accurate ARG-host attribution in metagenomic disease-association studies.

The clinical relevance of resistome profiling may be more direct in immunocompromised populations, where invasive infections frequently originate from gut translocation^63,64^. Patients undergoing allogeneic haematopoietic stem cell transplant (allo-SCT) represent a well-characterised example, with high rates of bloodstream infection (BSI) during neutropenia^65,66^. While microbiome composition has been associated with adverse outcomes such as graft-versus-host disease (GvHD), infection risk, and mortality^67,68^, resistome-focused analyses remain limited.

Available data suggest that antibiotic exposure drives expansion of the gut resistome in allo-SCT recipients, particularly following use of anaerobe-active agents^69,70^. In one study, increased ARG abundance and emergence of multidrug-resistance determinants were observed in patients who developed acute GvHD^71^, although limited ARG-host attribution constrains interpretation. These findings highlight the need for approaches that can resolve ARG context and host association in complex microbial communities, including emerging methods that integrate enrichment strategies with long-read sequencing.

Evidence linking gut resistome composition to infection risk is emerging. Metagenomic analyses have demonstrated that bloodstream infections can originate from strains present in the gut microbiome, with genomic concordance between stool and blood isolates^64^. These findings suggest that gut microbial and resistome composition may provide a basis for identifying patients at increased risk of invasive infection. Beyond these acute effects, longitudinal studies indicate that antibiotic exposure can induce sustained changes in resistome composition, with altered ARG and MGE profiles detectable months to years after treatment^72^. This indicates that even brief antibiotic exposures can result in prolonged perturbations of the resistome, with potential implications for recurrent infection risk and antimicrobial stewardship. Together, these observations support the concept that longitudinal resistome profiling could contribute to infection risk stratification and monitoring in high-risk clinical settings, although its clinical utility remains to be established.

However, several challenges limit the clinical interpretation of resistome profiling. Detection of ARGs does not necessarily imply phenotypic resistance, and distinguishing transient carriage from stable colonisation remains difficult. In addition, resistome signals are frequently confounded by underlying shifts in microbial community structure. Addressing these limitations will require approaches that integrate accurate host attribution, longitudinal sampling, and functional data to distinguish expressed from silent ARGs. Emerging multi-omic strategies, including metatranscriptomic and metaproteomic profiling, may help bridge the gap between genotype and phenotype, although their application in clinical settings remains limited.

Clinical translation will require interpretative frameworks that contextualise resistome findings alongside conventional microbiology, prioritising ARGs with established genotype-phenotype relationships, longitudinal persistence, and MGE association. Prospective studies in allo-SCT and other high-risk populations, including solid organ transplant, intensive chemotherapy, and critical care settings, will be required to determine whether resistome-derived metrics improve clinical decision-making without unintended consequences for antimicrobial prescribing that could drive the emergence of resistance.

#### Editing the gut resistome as a therapeutic intervention

Modulating the gut resistome may offer a means of reducing the burden of AMR in high-risk clinical settings, including haematopoietic stem cell transplantation, solid organ transplantation, and oncology. Interventions that reduce the abundance of resistant organisms, limit the spread of ARGs, or restore microbiome-mediated colonisation resistance may therefore influence the risk of resistant infections.

Resistome modulation strategies can be broadly categorised into three groups: (1) dietary interventions (including probiotics, prebiotics, and synbiotics), (2) intestinal microbiota transplantation (IMT), and (3) targeted editing using engineered bacteriophages.

Gut resistome profiling provides a framework to evaluate microbiota-targeted interventions by capturing their effects on ARG abundance, genetic context, and transmission potential.

##### Dietary and probiotic interventions

Experimental and clinical studies suggest that modulation of the gut microbiota can influence colonisation resistance and resistome composition. For example, defined commensal consortia can suppress multidrug-resistant Enterobacteriaceae through ecological competition, highlighting the role of microbial community structure in constraining expansion of resistant organisms^73^. Similarly, dietary composition has been associated with variation in ARG abundance and Enterobacteriaceae prevalence, suggesting that host and environmental factors can indirectly shape the resistome^74,75^.

Resistome profiling enables these effects to be quantified across both pathogenic and commensal taxa. Probiotic interventions, for instance, have been associated with reduced ARG burden and decreased prevalence of MDROs in some settings, although effects remain variable across studies and clinical contexts^76,77^. By resolving ARGs alongside their host and MGE context, mNGS provides a means to assess not only changes in abundance but also the potential for onward transmission.

Importantly, resistome profiling also enables evaluation of intervention safety. Some probiotic strains used in supplements and fermented products harbour ARGs, including those located on MGEs^78,79^, underscoring the need to detect potential risks, particularly in vulnerable populations. Integration of resistome profiling with detailed metadata, including diet, co-medications, and antimicrobial exposure, will be essential to distinguish direct intervention effects from broader ecological shifts.

Key uncertainties include the durability of intervention effects and their impact on non-dominant taxa. High-resolution mNGS, including ARG host attribution and MGE association, will be needed to evaluate these interventions’ effects on transmission-relevant resistance reservoirs.

#### Intestinal microbiota transplant - IMT

IMT, the transfer of stool from a screened donor to a recipient’s gut, is well established for recurrent *Clostridioides difficile* infection^80^ and is under investigation for other conditions, including inflammatory bowel disease^81^ and allo‑SCT–associated complications^82,83^. Some observational studies and early interventional data suggest that IMT may reduce colonisation with carbapenemase‑producing Enterobacterales and decrease gut ARG burden in selected high-risk populations^84–86^, though decolonisation rates are variable. However, findings are inconsistent, and in some cases IMT has been associated with acquisition of new ARGs and pathogens, including Shiga toxin‑producing and uropathogenic *E. coli* from donor material^87,88^.

Metagenomic resistome profiling enables quantification of these risks and provides mechanistic insight into how IMT remodels the gut microbiome. In a randomised, placebo-controlled trial in haematology patients, Rashidi et al.^89^ demonstrated a biphasic resistome response with transient transfer of low-abundance, donor-derived ARGs followed by durable resistome stabilisation and restoration of colonisation resistance. Similarly, deep mNGS studies in recipients of standardised microbiota therapeutics have shown sustained reductions in ARG richness and high-risk β-lactamase genes, coupled with recovery of Bacteroidetes and Clostridia populations^90^. These findings highlight the value of mNGS for identifying microbiological correlates of IMT success and for monitoring donor-derived ARG transmission over time. Integration of metabolomic data with mNGS may further clarify the mechanisms underlying IMT efficacy, particularly those linking community structure and resistome dynamics to metabolite-mediated effects, including short‑chain fatty acid production, which has been implicated in colonisation resistance against Enterobacteriaceae^91^.

Broader translation is currently limited by heterogeneity in donor material, antibiotic preconditioning, and analytical methods. Standardised mNGS workflows incorporating resistome endpoints, alongside systematic donor resistome screening, will be needed to define reproducible biomarkers of IMT efficacy and safety, and to minimise transmission of mobile ARGs.

#### Phage-based precision editing

Bacteriophage-based approaches may offer a targeted strategy for resistome editing. Engineered phages equipped with CRISPR–Cas systems have demonstrated proof-of-concept in vitro and in vivo, enabling selective killing of antibiotic-resistant *E. coli*, biofilm clearance, and reduction of intestinal colonisation in animal models^92^. In parallel, CRISPR-Cas constructs targeting specific resistance determinants, such as carbapenemase genes (e.g. *bla*KPC), have been shown to reduce resistance and partially restore antibiotic susceptibility in clinical Klebsiella isolates in vitro^93^, highlighting the potential for ARG-directed editing.

Resistome profiling can inform the design of these interventions by identifying dominant ARGs, their genomic context, and host taxa — parameters critical for phage selection and CRISPR guide design. Phage therapy nonetheless remains at an early translational stage, with key challenges including emergence of phage resistance, uncertain microbiome effects, regulatory complexity, and manufacturing constraints^94^. Longitudinal mNGS will be needed to monitor targeted ARG removal, detect compensatory resistance, and characterise off-target effects on commensal taxa.

### Conclusions and future horizons

Traditional methods for AMR surveillance and research have largely relied on culture-based susceptibility testing, targeted molecular diagnostics or WGS of individual bacterial isolates. While these approaches have provided important insights into specific resistance mechanisms and remain essential tools, they do not capture the broader dynamics of AMR within complex microbial communities. In contrast, metagenomic resistome profiling enables untargeted detection of ARGs, including those in difficult-to-culture organisms, and can resolve their genetic context, providing a more comprehensive view of resistance in vivo.

Metagenomic resistome profiling has evolved from the cataloguing of ARGs towards increasingly context-aware analyses that integrate genetic architecture, host attribution, and community-level dynamics. Despite this progress, key challenges remain, including accurately linking ARGs to their bacterial hosts, resolving genomic context within complex microbial communities, and determining whether detected ARGs are functionally expressed and contribute to phenotypic resistance. Advances in long-read sequencing and hybrid assembly have improved resolution of MGEs, while emerging approaches such as long-read targeted enrichment strategies, single-cell genomics, and spatially resolved metagenomics offer new opportunities to interrogate resistome structure at high resolution. In parallel, ML approaches are expanding the detectable resistome beyond known reference space. Together, these developments are driving a transition towards a more mechanistic understanding of how ARGs are maintained, mobilised, and transmitted, although continued integration of experimental and computational methods, standardisation of analytical frameworks, and alignment with clinically meaningful endpoints will be essential to realise this potential.

As sequencing technologies become more accessible and informatics capacity expands, integrating resistome analysis into clinical and public health settings can support earlier detection of resistance threats, inform antimicrobial stewardship, and enable more targeted interventions. By linking high-resolution resistome data with clinically relevant outcomes, these approaches have the potential to substantially advance how AMR is understood and managed, supporting more integrated and data-driven strategies to mitigate its global impact.
